# Arachidonic Acid Supplementation During Reproductive Aging Prevents Female Fertility Decline

**DOI:** 10.3390/ijms262311332

**Published:** 2025-11-24

**Authors:** Jie Tang, Yuanli Chen, Meng Zhou, Ling Zhao, Yanyan Li, Mingxue Li, Fengmei Yang, Zhanlong He, Longbao Lv

**Affiliations:** 1 Kunming Institute of Zoology, Chinese Academy of Sciences, Kunming 650201, Chinazhoumeng@mail.kiz.ac.cn (M.Z.); zhaoling@mail.kiz.ac.cn (L.Z.); 2 Faculty of Basic Medicine, Kunming Medical University, Kunming 650500, China; chenyuanli@kmmu.edu.cn; 3 State Key Laboratory for Conservation and Utilization of Bio-Resources in Yunnan, School of Life Sciences, Yunnan University, Kunming 650500, China; 4 Yunnan Key Laboratory of Vaccine Research and Development on Severe Infectious Diseases, Institute of Medical Biology, Chinese Academy of Medical Sciences & Peking Union Medical College, Kunming 650118, Chinalimingxue@imbcams.com.cn (M.L.);

**Keywords:** female reproductive aging, arachidonic acid, oocyte quality, lysosomal function, fertility intervention

## Abstract

Female reproductive aging precedes physical aging and is closely associated with the development of many aging-related diseases. With the delay in the human reproductive age, delaying reproductive aging and improving fertility have become important challenges for biomedical research. Arachidonic acid (AA) was found to significantly prolong the reproductive lifespan and enhance the quality of senescent oocytes in a cryptic nematode model. In a mouse model of senescence, AA supplementation improved the ovarian reserve, increased the oocyte number and quality, and restored fertility. Further analysis showed that AA delayed ovarian senescence by restoring lysosomal activity in the germ lines of senescent nematodes and mice and enhancing lysosomal functions in oocytes and ovarian granulosa cells. This study provides preliminary evidence that AA could serve as a potential intervention strategy for reproductive senescence and offers new ideas for improving reproductive health in older women.

## 1. Introduction

Female reproductive senescence is a multifaceted biological process characterized by a significant decline in the quantity and quality of oocytes with age [1], leading to reduced fertility and an increased susceptibility to undesirable pregnancy outcomes [2]. Due to the global population aging trend and delayed childbearing, addressing this issue is a major challenge in biomedical research [2]. Although the molecular mechanisms underlying reproductive aging are better understood, effective intervention strategies need to be developed, especially regarding nutritional interventions.

Reportedly, unsaturated fatty acids, especially arachidonic acid (AA), have attracted attention owing to their multifunctional roles in organisms. These unsaturated fatty acids are important energy sources. They also directly participate in follicle maturation and oocyte quality control by regulating fatty acid metabolism, alleviating oxidative stress, and optimizing signal transduction [3,4]. AA and other unsaturated fatty acids are significantly higher in mature follicular fluid than in immature follicular fluid in human females [5]. Additionally, studies have demonstrated the potential benefits of unsaturated fatty acids for reproductive health, including improvements in mammalian follicle development, oocyte maturation, and embryo quality [6,7] and enhanced reproductive performance in vertebrates [8,9] and invertebrates [10]. Although the role of unsaturated fatty acids in maintaining reproductive health is well known, their role in anti-reproductive aging mechanisms remains unclear. Specifically, AA exerts multiple effects on cellular physiological processes. For example, it regulates signal transduction pathways [11] and reduces inflammatory responses [12]. However, the ability of AA to directly affect the reproductive aging process, especially through potential cellular mechanisms, such as regulating lysosomal functions to delay the aging of oocytes and other reproductive cells, requires further investigation.

Lysosomes are the main digestion and waste-processing centers of cells and are responsible for breaking down aging, damaged, or dysfunctional proteins and organelles [13,14]. Autophagy, which is dependent on lysosomal functions [14], affects the quality and quantity of oocytes [15,16]. Defects in autophagy genes can lead to oocyte abnormalities during meiosis and increased DNA damage, further affecting reproductive health [17]. Although recent research has revealed the role of autophagy in reproductive health, mechanisms through which lysosomes regulate oocyte maturation and reproductive aging are underexplored. Lysosomal functions in oocytes [18] and the entire reproductive system [19] gradually decline with age, which may lead to a decreased reproductive capacity, and AA can activate autophagy [20] and lysosomal activity [20]. This study aimed to explore whether enhancing lysosomal function can effectively delay reproductive aging and evaluate the efficacy of AA as a potential intervention strategy for reproductive aging.

This study focused on the potential regulatory role of AA in reproductive aging, particularly how it improves oocyte quality and delays reproductive aging by influencing lysosomal functions. Our results showed that AA could significantly enhance lysosomal activity, thereby improving oocyte quality and delaying female reproductive aging. Findings from the current study broaden our understanding of intervention strategies for reproductive aging, providing a new biological basis and potential clinical application prospects for developing reproductive aging interventions based on AA.

## 2. Results

### 2.1. Unsaturated Fatty Acids Delay Reproductive Aging in Caenorhabditis elegans

**Figure 1:**
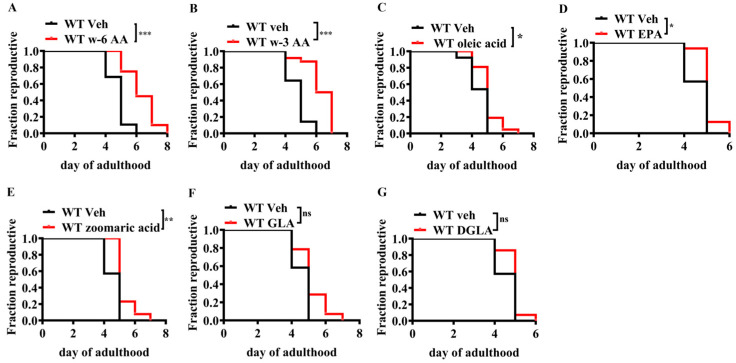
Effect of unsaturated fatty acids on the reproductive lifespan of *Caenorhabditis elegans* (*n* = 21–30). (**A**) ω-6 Arachidonic acid (ω-6 AA, 20:4, 10 µM) and (**B**) ω-3 arachidonic acid (ω-3 AA, 20:4, 10 µM) significantly extended the reproductive lifespan in *C. elegans*. (**C**) Oleic acid (18:1, 10 µM), (**D**) EPA (20:5, 20 µM), and (**E**) zoomaric acid (16:1, 10 µM) slightly extended the reproductive lifespan of *C. elegans*. (**F**) GLA (18:3, 10 µM) and (**G**) DGLA (20:3, 10 µM) had no significant effect on the reproductive lifespan. * *p* < 0.05, ** *p* < 0.01, *** *p* < 0.001; ns, no significant difference.

To validate the function of unsaturated fatty acids in reproductive aging, we used *C. elegans* as a model organism. *Caenorhabditis elegans* is an ideal model organism used to study reproductive aging [21,22,23,24], as genes that regulate reproductive aging in humans, mammals, and *C. elegans* are conserved [23,25,26,27,28]. Results showed that AA, oleic acid, zoomaric acid, and eicosapentaenoic acid delayed reproductive aging in *C. elegans* (Figure 1A–E), whereas gamma-linolenic acid and linoleic acid did not (Figure 1F,G). After a systematic screening of various unsaturated fatty acids, AA and other unsaturated fatty acids were found to significantly delay reproductive aging in *C*. *elegans*, with AA showing the most significant effect.

### 2.2. AA Improves Age-Induced Oocyte Quality in C. elegans

**Figure 2:**
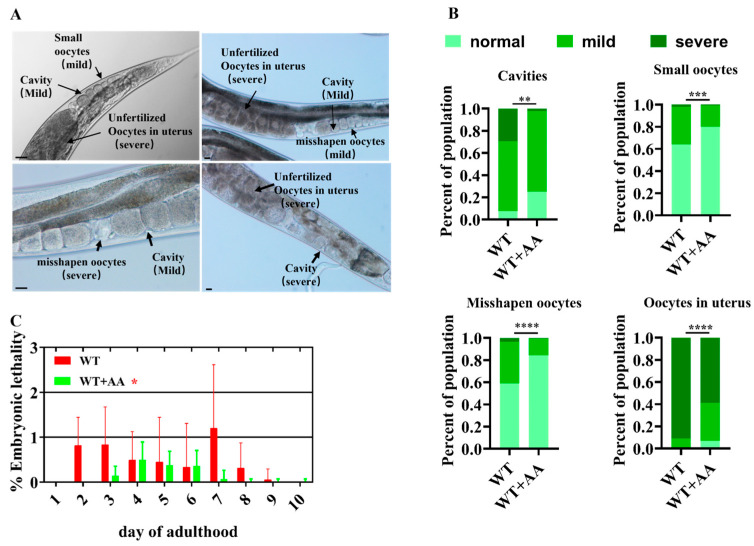
Arachidonic acid improves oocyte quality in aging *Caenorhabditis elegans*. (**A**) Representative images of oocyte morphological defects. Scale bar: 100 μm. (**B**) Oocyte morphological defect scores show that supplementation with ω-6 arachidonic acid significantly improves oocyte quality in 5-day-old worms (*n* = 135–165). (**C**) The addition of ω-6 arachidonic acid reduced the percentage of unhatched embryos produced by mated worms (n = 30). * *p* < 0.05, ** *p* < 0.01, *** *p* < 0.001, **** *p* < 0.0001.

Reproductive aging is mainly attributed to a deterioration in oocyte quality [1]. Therefore, we investigated the direct effect of AA on oocyte quality. AA supplementation significantly reduced the frequency of abnormal oocytes in reproductively senescent WT worms (Figure 2A,B) and improved embryo hatching rates (Figure 2C).

### 2.3. AA Delays the Decline in Fertility in Aging Mice

**Figure 3:**
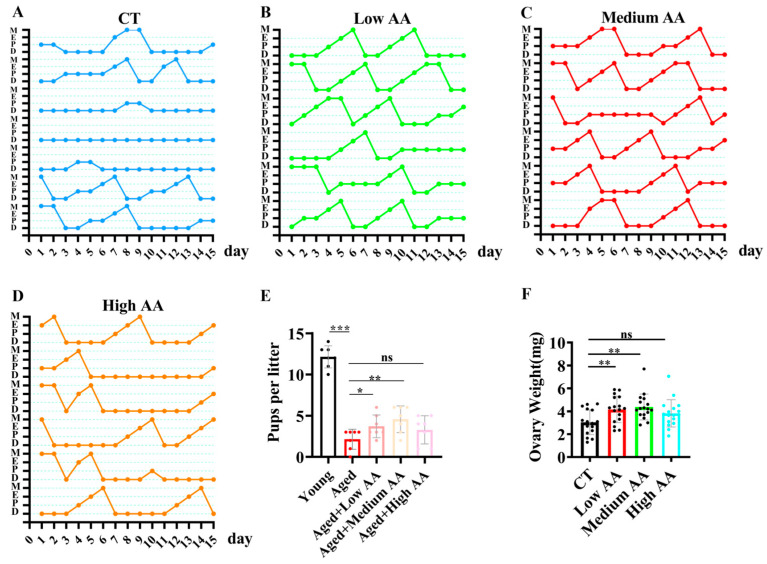
Arachidonic acid (AA) supplementation improves fertility in reproductive aging mice. (**A**–**D**) Estrous cycle patterns of 14-month-old mice in control and different AA-supplementation groups (*n* = 6), showing proestrus (P), estrus (E), metestrus (M), and diestrus (D). (**E**) Statistical analysis of litter size, with young mice at 3 months and aged mice at 14 months of age (*n* = 7). (**F**) Ovarian weights in 14-month-old mice (*n* = 15). * *p* < 0.05, ** *p* < 0.01, *** *p* < 0.001; ns, no significant difference.

Given the significant effects of AA in *C. elegans*, we explored its effects using a mammalian C57BL/6 mouse model to validate the evolutionary conservation of such properties. We explored the effects of three different AA concentrations on reproductive aging in mice. Vaginal imaging and mating detection were performed at 13 months of age after continuous AA feeding. Vaginal imaging revealed that the 36.5- and 75-mg/kg AA treatment groups had more regular estrous cycles than the aged control group (Figure 3A–D). The post-mating detection of litter sizes showed a significant decrease in the aged group compared to that in the young group. In contrast, supplementation with 36.5- and 75-mg/kg AA significantly rescued the age-induced decrease in the litter size (Figure 3E). Studies have shown that ovarian tissue undergoes atrophy as reproductive aging progresses, leading to a decrease in weight [29]. We found that supplementation with 36.5- and 75-mg/kg AA partially rescued the age-induced decrease in ovarian weight (Figure 3F).

### 2.4. AA Delays Follicle Loss and Ovarian Aging in Aging Mice

**Figure 4:**
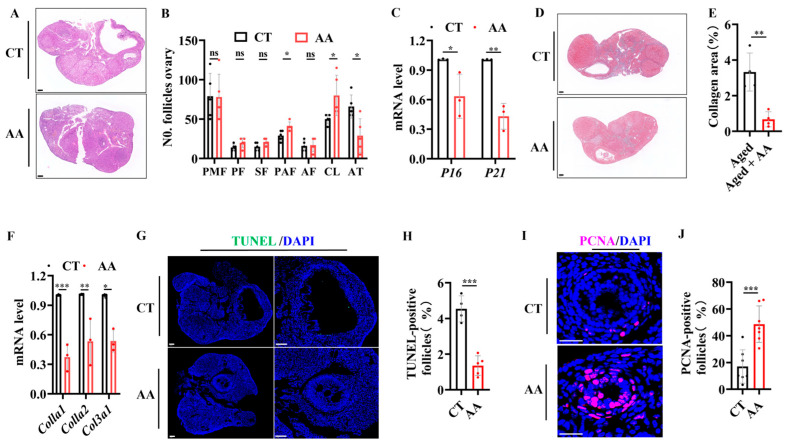
Arachidonic acid supplementation delays ovarian aging in mice. (**A**) Representative H&E staining images of ovaries from 14-month-old mice. (**B**) Statistical analysis of follicle development stages in ovaries of 14-month-old mice (*n* = 4), including the primordial follicle (PMF), primary follicle (PF), secondary follicle (SF), antral follicle (AF), preantral follicle (PAF), corpus luteum (CL), and atretic follicle (AT). (**C**) RT-PCR analysis of transcription levels of aging-related genes (*p16*, *p21*). (**D**) Representative Masson’s trichrome staining of ovaries from 14-month-old mice (*n* = 4). (**E**) Quantification of Masson’s trichrome staining. (**F**) RT-PCR analysis of transcription levels of fibrosis-related genes (*Col1a1*, *Col1a2*, *Col3a1*). (**G**) Representative TUNEL immunofluorescence images of ovaries from 14-month-old mice. (**H**) Quantification of TUNEL-positive granulosa cells. (**I**) Representative PCNA immunofluorescence images of ovaries from 14-month-old mice. (**J**) Quantification of PCNA-positive granulosa cells. Scale bar: 100 μm. * *p* < 0.05, ** *p* < 0.01, *** *p* < 0.001; ns, no significant difference.

To evaluate the effects of AA on the ovarian reserve and aging, we performed the following experiments. Considering the beneficial effect of 75 mg/kg AA, subsequent experiments were performed using this concentration. An evaluation of the effects of AA on the ovarian reserve was performed by staining ovarian tissue sections with hematoxylin and eosin (H&E). This showed an increase in preantral follicles and corpora lutea and a decrease in atretic follicles compared to numbers in age-matched controls (Figure 4A,B). The expression of the cell cycle-inhibitory proteins p16 and p21, which are markers of aging [29,30], was detected in the ovaries using real-time (RT)-PCR. mRNA expression in the AA-supplemented group was lower than that in the control group (Figure 4C). Fibrosis is a hallmark of ovarian aging and can affect oocyte release in the ovarian stromal region [31]. Masson’s trichrome staining of ovarian sections in mice showed a significant decrease in staining intensity in the AA group compared with that in the control group (Figure 4D,E). RT-PCR analysis of the fibrosis-related genes *Col1a1*, *Col1a2*, and *Col3a1* revealed significantly lower expression in the AA group than in the control group (Figure 4F). To study the cellular changes in ovarian granulosa cells, we used PCNA and TUNEL staining to detect granulosa cell proliferation and apoptosis during follicle development [29]. The results showed a significantly lower apoptosis rate (Figure 4G,H) and an increased proliferation rate (Figure 4I,J) in granulosa cells from the AA group compared to those in the control group. These results suggest that AA supplementation delayed age-related decreases in the ovarian reserve and senescence in mice.

### 2.5. AA Increases the Quantity and Quality of Oocytes in Aging Mice

**Figure 5:**
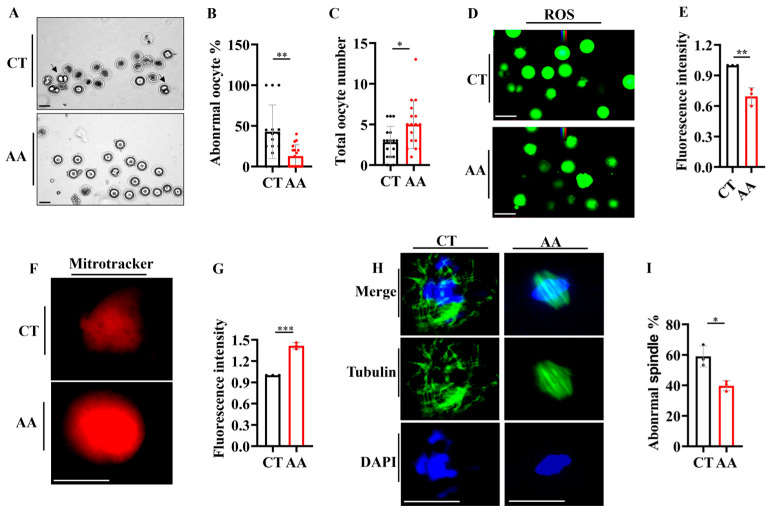
Arachidonic acid supplementation delays the decline in oocyte quality in mice. (**A**) Representative images of oocytes from 14-month-old mice. Black arrows indicate abnormal oocytes. (**B**) Statistical analysis of abnormal oocytes in 14-month-old mice. (**C**) Total oocyte counts in 14-month-old mice. (**D**) Reactive oxygen species (ROS) staining of oocytes from 14-month-old mice (*n* = 10–16 oocytes). (**E**) Quantification of ROS fluorescence staining from (**D**). (**F**) MitoTracker staining of oocytes from 14-month-old mice (*n* = 12–18 oocytes). (**G**) Quantification of mitochondrial fluorescence staining from (**F**). (**H**) Representative images of spindles and chromosome alignment of MII oocytes from control and arachidonic acid-supplemented 14-month-old mice (*n* = 16–21 oocytes). (**I**) The rate of abnormal spindles in MII oocytes from (**H**). (**D**–**I**) Oocyte fluorescence for each mouse was averaged and presented as a single data point; total oocyte number per group reflects all oocytes from three mice. Scale bar: 100 μm. * *p* < 0.05, ** *p* < 0.01, *** *p* < 0.001.

Significant improvements were observed during the study of the effects of AA on the quantity and quality of oocytes in aged mice. AA-supplemented mice showed a reduced rate of abnormal oocytes (Figure 5A,B) and the total number of ovulation events in 14-month-old mice (Figure 5C) compared to the control group. This shows that AA supplementation may play a positive role in preserving or improving the quality of oocytes.

To further explore the protective effect of AA on oocyte quality, we determined the levels of reactive oxygen species (ROS) using the ROS-sensitive fluorescent dye H2DCFDA. ROS levels were significantly reduced in oocytes from the AA-treated group compared to those in the control group (Figure 5D,E). In addition, we assessed the functions of mitochondria, a key organelle in energy production, in oocytes and found that their dysfunction was strongly associated with decreased oocyte quality [29,32]. Using MitoTracker staining, we found that the oocytes in the AA-treated group exhibited higher mitochondrial activity (Figure 5F,G), which further supports the positive role of AA in maintaining and improving oocyte functions. Finally, we assessed the spindle structure and chromosome arrangement via immunofluorescence [2], as these are key indicators used to assess oocyte quality. AA-treated mice exhibited a more complete and regular spindle structure and chromosomal arrangement during meiosis (Figure 5H,I), suggesting that AA may enhance oocyte quality by maintaining the integrity of the cell division machinery. Overall, our data strongly support the potential application of AA in enhancing the quantity and quality of oocytes in aged mice.

### 2.6. AA Delays Ovarian Aging by Enhancing Lysosomal Functions

**Figure 6:**
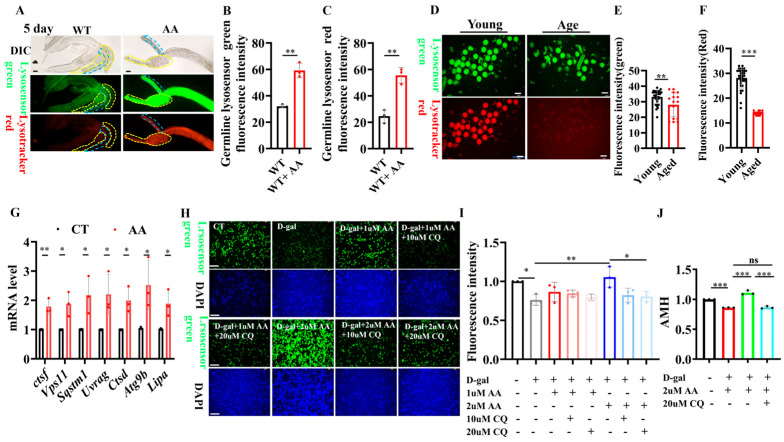
Arachidonic acid delays ovarian aging by enhancing lysosomal functions. (**A**) Representative images of lysosomal staining in *Caenorhabditis elegans* germline (*n* = 50’80). (**B**) Quantification of LysoSensor Green fluorescence in germline. (**C**) Quantification of LysoTracker Red fluorescence in germline. (**D**) Representative images of LysoSensor Green and LysoTracker Red fluorescence in oocytes from 3 and 14monthold mice (*n* = 16–32 oocytes). (**E**) Quantification of LysoSensor Green fluorescence in oocytes from 3- and 14-month-old mice. (**F**) Quantification of LysoTracker Red fluorescence in oocytes from 3- and 14-month-old mice. (**G**) qPCR analysis of lysosomal mRNA levels in ovaries from control and arachidonic acid-supplemented 14-month-old mice. (**H**) Representative images of LysoSensor Green staining in mouse ovarian granulosa cells treated with D-galactose for 48 h and supplemented with arachidonic acid or chloroquine. (**I**) Quantification of (**H**). (**J**) ELISA detection of anti-Müllerian hormone (AMH) levels in ovarian granulosa cell supernatant. Scale bar: 100 μm. * *p* < 0.05, ** *p* < 0.01, *** *p* < 0.001; ns, no significant.

Reportedly, AA activates lysosomal activity [20], and lysosomal activity plays an important role in reproductive aging [19]. We aimed to determine whether AA retards reproductive aging via lysosomes. AA supplementation rescued the age-induced reduction in lysosomal activity in the nematode germline, as demonstrated through LysoTracker and lysosome sensor staining assays (Figure 6A–C). We simultaneously assayed the lysosomal activity of oocytes from young and old mice and found that in old mice, it was significantly lower than that in young mice (Figure 6D–F). Lysosome-related gene activities in mouse ovaries were then examined via quantitative real-time PCR (qPCR). Here, the mRNA expression of lysosomal activity-related genes was elevated in AA-supplemented aged mice compared to that in controls of the same age (Figure 6G).

Furthermore, we used D-galactose to induce senescence in mouse ovarian granulosa cells. LysoTracker and LysoSensor staining were used to validate that AA delays ovarian senescence by activating lysosomal functions. AA supplementation significantly enhanced lysosomal activity in senescent ovarian granulosa cells. Furthermore, the lysosome-activating effect of AA was blocked when lysosomal inhibitors were added (Figure 6H,I). In addition, we examined anti-Müllerian hormone (AMH) levels in the supernatant of ovarian granulosa cells and found that D-galactose induction reduced AMH secretion in ovarian granulosa cells after 48 h. Meanwhile, the addition of AA rescued the reduction in AMH secretion induced by D-galactose. Furthermore, adding the lysosomal inhibitor chloroquine blocked the lysosome-activating effect of AA (Figure 6J). The central role of lysosomes in AA-mediated anti-ovarian aging was thus further reinforced. These results suggest that AA delays female reproductive senescence by enhancing lysosomal functions.

## 3. Discussion

This study validated the role of polyunsaturated fatty acids, particularly AA, in delaying reproductive aging and provides new insights into this field. Our results revealed that AA directly improves oocyte quality by activating lysosomal activity, potentially delaying reproductive aging mechanisms. These findings align with those of previous studies, where polyunsaturated fatty acids were reported to have potential benefits for reproductive health, including supporting oocyte maturation, optimizing embryo development, and maintaining endometrial receptivity [3,9]. These findings provide a new intervention strategy for delaying female reproduction-related cell and tissue senescence, with the potential for further development as a therapeutic strategy.

Our study further confirms the central role of lysosomes in regulating reproductive senescence, which aligns with the findings of Bohnert and Kenyon in *C. elegans* [33]. Lysosomes act as cellular “recycling centers” and are crucial for degrading damaged organelles and proteins to maintain cellular stability and function [33]. These findings provide new insights into how unsaturated fatty acids regulate germ cell mass and longevity at the molecular level. Although the specific role of lysosomes in reproductive senescence is not fully understood, our data suggest that enhanced lysosomal activity is directly associated with improved oocyte quality and a prolonged reproductive lifespan. This was verified in the *C. elegans* model [19] and mammalian models [15,16]. This suggests that lysosomal activity may be conserved across species to regulate germ cell quality and function.

Studies have shown that lysosomal biogenesis, signaling, and function are regulated by a highly integrated network involving dozens to hundreds of regulatory genes and multiple interconnected pathways [34]. For example, the transcription factors TFEB [35] and USF2 can respectively activate or repress broad lysosome-related gene programs and interact with mTORC1, chromatin remodeling complexes, and other metabolic sensors to mediate adaptive cellular responses. In Caenorhabditis elegans, multiple longevity mutants including *daf-2*, *isp-1*, and *eat-2* not only delay aging [36] but also extend reproductive lifespan [27]. More than 40 lysosomal genes have been found to be influenced by multiple longevity pathways during aging [36]. Given this complexity, our current work focused on the physiological contribution of arachidonic acid–lysosome interaction, and, due to feasibility constraints, did not perform a systematic genetic or pathway analysis of this network. Therefore, we acknowledge this limitation and propose that future mechanistic research should employ genetic and molecular approaches to identify the key lysosomal regulatory nodes mediating AA’s beneficial effects on reproductive aging.

Recent studies have highlighted new molecular mechanisms by which polyunsaturated fatty acids (PUFAs) and their derivatives modulate aging and cell senescence beyond simple nutritional benefits. For example, Folick et al. [37] demonstrated that lysosome-derived bioactive lipids, such as arachidonic acid, act as signaling molecules that relay information from lysosomes to the nucleus. These signals regulate transcriptional programs for longevity through nuclear hormone receptors and lipid chaperones in Caenorhabditis elegans, establishing lysosomal fatty acid signaling as a pivotal regulator of germline and somatic aging. Recent research in mammalian cells further revealed that epoxyeicosatrienoic acids (EETs), metabolites of arachidonic acid, can alleviate cellular senescence and oxidative stress injury via the Trim25/Keap1/Nrf2 axis [38]. Mechanistically, EETs promote Trim25-mediated ubiquitination and degradation of Keap1, thus allowing Nrf2 to translocate into the nucleus and upregulate antioxidant defenses. This regulatory process suppresses endoplasmic reticulum stress and senescence-associated gene expression in aging cells, providing a cross-species link between fatty acid metabolism and cellular stress response pathways. Together, these advances strongly encourage investigation of arachidonic acid and related PUFAs as active interventions to delay reproductive aging and counteract cellular decline. Mechanistic and translational studies targeting these lipids signaling pathways may yield new strategies for maintaining reproductive and systemic health.

Furthermore, despite the many advances in our research, several limitations remain. First, although nematode and mouse models provide useful biological insights, the complexity of human reproductive aging may limit their direct applications. Therefore, translating these findings to clinical applications in humans requires further investigation and validation. Second, in a pre-stage study of concentration mapping in nematodes, it was found that nematodes were unable to produce offspring at high doses (50 mM) of arachidonic acid exposure. Therefore, studies on the optimal dose of AA, the safety of long-term supplementation, and potential side effects are limited. Finally, the reproductive health-promoting effects of AA may be related to an individual’s metabolic status, genetic background, and overall dietary patterns. These issues need to be addressed in future studies. In conclusion, our study provides a preliminary scientific basis for using AA as an intervention strategy to address reproductive aging. Future research should explore its mechanisms of action and evaluate its potential clinical applications, especially in improving reproductive health and fertility in older women.

## 4. Materials and Methods

### 4.1. Reproductive Span Analysis of C. elegans

The reproductive span of the organism was determined following the method described previously [27]. The reproductive span was determined by transferring parental nematodes to fresh medium daily until offspring production ceased after 2 days. This was designated as the first day of viable offspring production until no offspring were observed.

### 4.2. Oocyte Morphology Experiment Using C. elegans

The oocyte morphology was analyzed as previously stated [23]. On day 5 of nematode reproduction, each nematode oocyte image was scored. These images included four deteriorating phenotypes (cavities, small oocytes, malformed oocytes, and intrauterine oocytes) and three levels of severity (mild, moderate, and severe) [39]. In summary, each category received a rating of normal, mild, or severe according to the extent of the observed phenotype. For the ‘cavities’ category, normal meant cavity absence, mild identified partial separation among oocytes, and severe described prominent gaps between oocytes. In the ‘misshapen oocytes’ group, normal reflected uniformly shaped oocytes (mostly cuboidal), mild indicated minor shape irregularities, and severe involved oocytes exhibiting pronounced deformation or damage. For the ‘small oocytes’ criterion, normal denoted all oocytes of expected size, mild signified incomplete occupancy of the space between the body wall and germline arm, and severe represented the presence of notably undersized oocytes. Within the ‘oocytes in uterus’ category, a normal rating indicated that only embryos were detected in the uterus, mild signified the presence of at least one visible unfertilized oocyte, while severe corresponded to finding several separate unfertilized oocytes or a cluster thereof inside the uterus. The images were scored according to the previously described criteria [39].

### 4.3. Animal Husbandry and Treatment

Female C57/BL6 mice (12 months old) were obtained from the Institute of Medical Biology, Chinese Academy of Medical Sciences. The mice were housed under a 12 h light/dark cycle with a temperature range of 22–25 °C. The mice were randomly divided into four groups: one group served as the control and was administered a 1% sodium carboxymethyl cellulose (MackLin, Shanghai, China, lot#C14474187) solution via gavage. The remaining three groups were supplemented with AA (MackLin) at concentrations of 36.5 mg/kg/day (Low), 75 mg/kg/day (Medium), and 150 mg/kg/day (High) via gavage. AA at the three concentrations was prepared in a 1% sodium carboxymethyl cellulose solution and administered via gavage every other day. After the 2-month treatment period, mice were randomly selected for breeding, and the litter size was recorded to assess the reproductive capacity. Other mice were used to evaluate follicular retention, oocyte quantity and quality, lysosomal activity, and age-related gene expression. Eight-week-old mice served as the young control group. The animal experiments were approved by the Ethics Committee of the Institute of Medical Biology, Chinese Academy of Medical Sciences & Peking Union Medical College, with approval number DWSP202207002.

### 4.4. Collection of Oocytes

Female mice were administered 5 IU of pregnant mare serum gonadotropin (Solarbio, Beijing, China) intraperitoneally, followed by 5 IU of human chorionic gonadotropin (Solarbio, Beijing, China) 46–48 h later to induce superovulation. After 14–16 h, the mice were euthanized, and the ovaries were removed. Cumulus-oocyte complexes (COCs) were obtained from the ampullary region of the oviduct and cultured in an M2 medium (Nanjing Aibei Biotechnology, Nanjing, China). The COCs were briefly exposed to M2 medium containing 1% hyaluronidase (Solarbio, Beijing, China) to remove the cumulus cells.

### 4.5. Measurement of ROS Levels in Oocytes

Cells were stained with the H2DCFDA fluorescent dye (10 µmol/L, Invitrogen, Carlsbad, CA, USA) to detect ROS in oocytes for 30 min and photographed under a fluorescence microscope.

### 4.6. Fluorescence Experiment to Detect Mitochondrial Distribution in Oocytes

To determine the mitochondrial distribution in oocytes, oocytes from each group were cultured in an M2 medium containing 200 nM MitoTracker Red (Invitrogen, Waltham, MA, USA) at 37 °C with 5% CO_2_ for 2.5 h. Subsequently, the oocytes were washed three times with M2 medium and observed under an optical microscope (Leica, Wetzlar, Germany). The fluorescence intensity was calculated using ImageJ software (ImageJ2/1.54f).

### 4.7. Immunofluorescence Staining

Oocytes were fixed in a 4% paraformaldehyde solution for 20 min, followed by permeabilization with 0.5% Triton X-100 in phosphate-buffered saline (PBS) for 20 min. After several washes with PBS, oocytes were blocked overnight in a blocking buffer (1 × PBS/5% BSA/0.3% Triton™ X-100). Subsequently, oocytes were incubated with the diluted primary antibody (antibody dilution buffer: 1× PBS/1% BSA/0.3% Triton™ X-100) at 4 °C. CST was used as the primary antibody (a-Tubulin (11H10) Rabbit mAb Alexa Fluor 488 conjugate, #5063) to visualize the spindle apparatus at a dilution of 1:400. After rewashing with PBS, oocytes were stained with 10 μg/mL propidium iodide for 10 min, mounted on slides, and imaged using a fluorescence microscope.

### 4.8. Determination of AMH Levels

The ovarian granulosa cell supernatant was collected. AMH and sex hormone levels were measured in each group of supernatants using a chemiluminescent immunoassay kit (Cusabio, Houston, TX, USA) following the manufacturer’s instructions.

### 4.9. Estrous Cycle Monitoring

Vaginal smears were collected from mice for 15 consecutive days. Smears were stained with H&E and evaluated under a microscope (Leica). Estrous cycle stages were determined following previously described criteria [40].

### 4.10. qPCR

Total RNA was extracted from ovaries using the TRIzol method. Subsequently, qPCR was performed using the one-step method following the instructions of TaKaRa’s One-Step TB Green PrimeScript PLUS RT-PCR Kit (cat#RR096A). Relative gene expression levels were determined based on a comparison with *gapdh* expression levels.

### 4.11. Ovarian Follicle Quantification

The ovaries were fixed in 4% paraformaldehyde, dehydrated and embedded in paraffin, and then cut into 8 μm-thick sections for staining with H&E. Follicle quantification was performed as described previously [16,41]. Briefly, follicles were scored every five sections (40 μm apart), counting only follicles with clear oocyte nuclei in each section and multiplied by 5 to calculate the total number of follicles in the ovary. According to the criteria established by Peterson and Peters 48, ovarian follicles at different developmental stages can be distinguished as follows (Table 1):

### 4.12. Masson’s Staining for Ovarian Fibrosis

Fibrosis is the hallmark of ovarian aging. Masson’s staining is one staining method used to demonstrate tissue fibrosis, where collagen fibers appear blue. This was assessed following the steps of the staining kit (Solarbio, Modified Masson’s Trichrome Stain Kit, G1346); after neutral resin sealing, the sample was allowed to air-dry naturally before scanning.

### 4.13. Immunofluorescence and Immunohistochemical Staining of Ovarian Tissues

Following the manufacturer’s instructions (Servicebio, Wuhan, China), TUNEL and PCNA immunofluorescence staining was performed using the TUNEL and PCNA detection kits. TUNEL-positive shows green fluorescence, PCNA-positive shows red fluorescence, and DAPI shows blue fluorescence.

### 4.14. Ovarian Granulosa Cell Culture and Treatment

Confluent granulosa cells were resuspended in medium composed of 10% fetal bovine serum (FBS; Gibco, Waltham, MA, USA), 1% penicillin-streptomycin (Beyotime, Haimen, China; C0224), and 90% DMEM (without sodium pyruvate; Gibco, C12430500BT). Cells were seeded into 48-well plates at a density of 1 × 10^5^ cells per well and incubated at 37 °C in a humidified incubator with 5% CO_2_. To induce cellular senescence, granulosa cells were treated with 20 g/L D-galactose. Simultaneously, different concentrations of arachidonic acid (AA) were added as indicated. Where applicable, the lysosomal inhibitor chloroquine was added during AA treatment. After 48 h of incubation, cell culture supernatants were collected for subsequent analyses, and the cells were used for lysosome staining. For each experiment, data from three replicate wells were averaged as one technical repeat, and three independent biological experiments were performed.

### 4.15. Lysosome Staining

Lysosomal staining of *C. elegans* was performed as previously described [19]. After seeding, ovarian granulosa and stromal cells were treated with 20 g/L D-galactose for 24 h to induce senescence. LysoTracker Red DND-99 (Invitrogen, Waltham, MA, USA) and LysoSensor Green DND-189 (Invitrogen) were added at a final concentration of 50 nM each. The cells were then incubated in a 37 °C cell culture incubator for 30 min. The cells were washed three times with PBS, and fluorescence was observed under a fluorescence microscope.

### 4.16. Data Analysis

Statistical analyses were performed with GraphPad Prism, version 10.1.2 (GraphPad Software Inc., La Jolla, CA, USA). For comparisons involving multiple groups, a one-way analysis of variance (ANOVA, San Francisco, CA, USA) was applied, followed by *t*-tests for specific pairwise comparisons. Reproductive span was represented by Kaplan-Meier curves, and the statistical significance of differences between groups was determined by the log-rank test. In all tests, results were deemed statistically significant when the *p*-value < 0.05.

## Figures and Tables

**Table 1:** Table of Developmental Characteristics of Oocytes at Different Stages.

Primordial follicle	Small oocyte surrounded by no attached cells or a single layer of flat pregranulosa cells.
Primary follicle	Medium-sized oocyte surrounded by alternating layers of single columnar granulosa cells.
Secondary follicle	Large oocyte surrounded by a transparent zone and more than two layers of cuboidal granulosa cells, enclosed within the basement membrane, known as a secondary follicle; at this stage, the antrum begins to form.
Antral follicle	Enlargement of the antrum, formation of cumulus structure, and late primary oocyte reaching maximum diameter.
Mature follicle	Follicle occupying the entire ovarian cortex and protruding towards the ovarian surface, the dominant follicle matures, and the antral follicle becomes sealed.
Atretic follicle	The oocyte nucleus shrinks, the chromosomes and cytoplasm dissolve, the granulosa cell layer undergoes apoptosis and decreases, the follicular membrane cells enlarge, and lipid-like substances appear in the cytoplasm, become luteinized and disperse in the connective tissue.

## Data Availability

The original contributions presented in this study are included in the article. Further inquiries can be directed to the corresponding authors.
